# ASSOCIATIONS BETWEEN PERSONALITY TRAITS AND COGNITIVE RESILIENCE IN OLDER ADULTS

**DOI:** 10.1093/geroni/igz038.2860

**Published:** 2019-11-08

**Authors:** Eileen K Graham, Bryan James, Daniel K Mroczek

**Affiliations:** 1 Northwestern University, Chicago, Illinois, United States; 2 Rush University, Chicago, Illinois, United States

## Abstract

There are considerable individual differences in the rates of cognitive decline across later adulthood. Personality traits are one set of factors that may account for some of these differences. The current project explores whether personality traits are associated with trajectories of cognitive decline, and whether the associations are different before and after a diagnosis of dementia. The data will be analyzed using linear mixed effects regression. Across these goals is a focus on replicability and generalizability. Each of these questions will be addressed in four independent longitudinal studies of aging (EAS, MAP, ROS, SATSA), then meta-analyzed, thus providing an estimate of the replicability of our results. This study is part of a registered report of existing data that is currently under stage 1 review.

